# Duration of Action of Antidotes of the Principal Remedies

**Published:** 1890-10

**Authors:** F. H. Lutze

**Affiliations:** Cheshire, New York


### DURATION OF ACTION AND ANTIDOTES OF THE PRINCIPAL REMEDIES.

F. H. LUTZE, M. D., CHESHIRE, NEW YORK.

When first I saw in Hahnemann's *Chronic Diseases* that a single dose of the homœopathic remedy would often act for from six hours to eight weeks and even three months, and afterward found this to be actually so from personal experience, it occurred to me that it would perhaps benefit others who did not have access to that work, to know this fact, and deter them from repeating the remedy too often, as I rather think is customary with many homœopaths. The following compilation is the result.

I am aware that the duration of the action of a remedy is by no means positively always the same, but depending somewhat upon the nature of the disease, whether acute or chronic, and also upon the idiosyncrasy of the patient, yet hope that the knowledge that Hepar-s-c., for example, has been found to act for eight weeks and longer, may be of some material benefit to many a young homœopath as well as his patients. For it is a positive fact that many a case is spoiled by repeating the remedy too often instead of allowing one dose to continue its beneficent curative action to the end. Hahnemann's *Chronic Diseases*, Dr. C. von Bœnninghausen's works, and Hering's condensed *Materia Medica* have been used in collecting the data for the following list of

### THE MOST FREQUENTLY USED REMEDIES OF THE HOMŒOPATHIC MATERIA MEDICA, THEIR DURATION OF ACTION, ANTIDOTES, AND COMPLEMENTARY AND INIMICAL REMEDIES.

Those remedies which are taken from Hahnemann's *Chronic Diseases* and in the older works are called "Antipsorics," in this list are designated by heavy black letters. Those which in the older works are also called antipsorics, but which are doubtful, in this list appear in *italics*.

*Acetic acid.*

Antidotes: Lime-water, Magnes., Calc-c., Natrum-mur.

Acetic acid antidotes: All anæsthetic vapors, Acon., Asar., Coffea, Euphorb., Hepar, Ignatia, Op., Stram., Tabac., Alcohol.

Complementary: China in hemorrhages.

Inimical: Borax, Caust., Nux-v., Ranuncul-bulb., Sarsapar.

Aconitum-nap. Acts six to forty-eight hours.

Antidotes: Acetic acid., Paris, Vinum.

Acon. antidotes: Bell., Cham., Coff., Nux-v., Petrol., Sep., Sulph., Veratrum-alb.

Complementary to: Arn., Coff., Sulph. (high).

**Agar-musc.** Acts forty days.

Antidotes: Charcoal, Coffee, Wine, Brandy, Camphor, Fat, or Oil; Calc-c., Puls., Rhus-tox.

Agnus-castus. Acts eight to fourteen days.

Antidotes: Camph., Natr-mur.

Ailanthus-gland.

Antidotes: Aloe, Rhus-tox., Nux-v.

Nervous sensitive persons; bilious temperament, stout, and robust.

*Aloe-soc.*

Antidotes: Sulph., Mustard, Camph., Nux-vom., Lycopod.

Old people, phlegmatic and indolent persons.

Aloe has many symptoms like Sulphur, and is equally important in chronic diseases, with abdominal plethora.

**Alumina.** Acts over forty days.

Antidotes: Bry., Camph., Cham., Ipec.

Alumina antidotes lead poisoning.

Complementary: Bryonia-alb.

Constipation of infants; stools green, acidity of primæ viæ; Puberty: Chlorosis, with longing for indigestible substances.

Dark complexion, excitable. Mild disposition. Lack of animal heat; spare habit. Old people, hypochondriacal.

*Ambra grisea.* Acts for three to five weeks.

Antidotes : Camph., Coff., Nux-v., Puls., Staphis.
Ambra antidotes : Staphis., Nux-v.

**Ammon-carb.** Acts over thirty-six days.
Antidotes : Arnica, Camph., Hepar, vegetable acids and fixed oils, as : Olive, Castor, Linseed.
Amm-c. is an antidote to: Rhus poisoning and stings of insects.
Inimical to Lachesis.

**Ammon-mur.** Acts over six weeks.
Antidotes : Camph., Coff., Nux-v.

**Anacardium orient.** Acts over thirty days.
Antidotes: Coff., Juglans; for the anger and violence of mind : smelling of raw coffee. (Gastric and nervous disorders during pregnancy ; nervous and hysterical females.)

*Angustura.* Acts three to four weeks.

*Antimon-crud.* Acts four weeks.
Antidotes : Calc-c., Hepar-s-c., Mercur.
Anti-crud. antidotes : Stings of insects.
Complementary : Squilla.

*Antimon-tart.* Acts two weeks.
Antidotes : Asafœt., China, Coccul., Ipec., Laurocer., Opium, Puls., Sep.
Anti-tart. antidotes : Sepia.

Apis-mel.
Antidotes : Natr-mur., Ipec., Laches., Lact-ac. Apis high ; Salt, sweet oil, onions.
Apis antidotes : Cantharis, China, Digital.
Complementary : Natr-mur.
Inimical : Rhus-tox.

*Argent-met.* Acts two to three weeks.
Antidotes : Mercur., Puls.

Argent-nitr.
Antidotes : Natr-mur., Ars., Milk, Calc-c., Puls., Sepia, Lycopod., Merc., Silicea, Rhus-tox., Phos., Sulph.
Arg-nit. antidotes : Ammon-caust.
Melancholy : Congestions to head and chest, epistaxis, climaxis, flushes, itching skin.

Arnica-mont. Acts two to six days.

Antidotes: Camph., Ipec., Acon., Ars., China, Ignat.

Arn-mont. antidotes: Ammon-carb., China, Cicuta, Fer., Ignat. Ipec. Seneg.

**Arsenicum-alb.** Acts over thirty-six days.

Antidotes to large doses: Sesquioxide of iron, hydrated peroxide of iron, precipitated carbonate of iron, juice of sugar cane or honey-water. Lime-water in copious draughts, emetics of sulphate of zinc. Carbonate of potash and magnesia shaken in oil; Infusion of astringent substances.

Antidotes to small doses: Camphor, Chin., Chin-sulph., Fer., Hepar, Iod., Ipec., Nux-v., Sambuc., Tabac., Veratr.

Ars. antidotes: Carb-v., China, Fer., Graph., Iod., Ipec., Lach., Merc., Nux-v., Veratr., Lead poisoning and evil effects of alcohol.

Hydrogenoid constitution of Grauvogl, complaints of drunkards.

Arum-triph.

Antidotes: Buttermilk; Lactic acid.

*Asafœtida.* Acts four to six weeks.

Antidotes: Camph., Caust., China, Puls., Merc., Valerian.

Phlegmatic temperament; scrofulous, bloated, clumsy children, Venous, hemorrhoidal constitution, nervous people; syphilitics, who have taken much Mercury.

Asarum-europ. Acts eight to fourteen days.

Antidotes: Camph., Vinegar, and all vegetable acids. Nervous temperament, excitable or melancholic.

**Aurum-met.** Acts over six weeks.

Antidotes: Bell., Chin., Coccul., Coff., Cupr., Merc., Puls., Spigel., Sol-nig.

Aurum antidotes: Merc., Spigel.

Girls at puberty; old people, weak vision. Sanguine temperament; scrofulous, syphilitic, and mercurial patients.

**Baryta-carb.** Acts forty to fifty days.

Antidotes: Ant-tart., Bell., Camph., Dulc., Zincum.

Old fat people; scrofulous children, dwarfed in body and mind; general emaciation.

**Belladonna** acts over five weeks.
Antidotes: Coffea, Hyos.; Camph., Hepar, Opium, Puls., Vinum. Vinegar increases the headache.
Bell. antidotes: Acon., Cupr., Fer., Hyos., Mercur., Plumb.; Jaborandi?
Complementary: Calc-carb.
Bell. suits plethoric, lymphatic constitutions, who are jovial and entertaining when well, but irritable and violent when sick. Women, children, blue eyes, light hair, fine complexion, delicate skin.

Benzoic acid.
It antidotes Copaiba.
Rheumatic or gouty diathesis; especially in syphilitic or gonorrhœal patients.

Berberis-vulg.
Antidotes: Camph.
Berb. antidotes: Acon.

*Bismuthum.* Acts five to seven weeks.
Antidotes: Cal-c., Caps., Nux-v.

**Borax.** Acts seven to eight weeks.
Antidotes: Cham., Coff.
Inimical to Borax: Acetum, Vinum.

**Bovista.** Acts for fifty days.
Antidote: Camphor.
Coffee disturbs its action.
Palpitation in old maids, stammering in children.

*Bryonia-alba.* Acts two to three weeks.
Antidotes: Acon., Alum., Camph., Cham., Clemat., Coff., Ignat., Mur-ac., Nux-v., Puls., Rhus-tox., Senega.
Bry. antidotes: Rhus-t., chlorine.
Similar: Colocynth.
Complaints: from warm weather following cold days; exposure to heat of fire.

Cactus-grand.

Antidotes: Acon., Camph., China.

*Caladium.* Acts six to eight weeks.
Antidotes: Camph., Caps., juice of sugar cane.

Calad. antidotes: Mercur.
Complementary: Nitr-ac.

**Calc-carb.** Acts over fifty days.
Antidotes: Camph., Nitr-acid., Nitr-spr-dulc., Nux-vom., Sulph.
Calc. antidotes: Acet-ac., Bismuth., Chin., Chin-sulph., Nitr-ac.
Complementary to Calc.: Bell.
Leuco-phlegmatic temperament; fair, plump children, with open fontanelles and sutures, excessive obesity of young people.

Camphora. Acts five to fifteen minutes.
Antidotes: Opium. Nitr-spr-dulc., Dulcamara.
Camph. antidotes: Canth., Cupr., Squilla.
Inimical: after Nitrum.

Cannabis-sativa. Acts two to three weeks.
Antidote: Camph., Lemon juice.

*Cantharis* acts three weeks.
Antidotes: Acon., Camph., Lauroceras., Puls. Oil increases the pernicious effects of Cantharis.
Inimical: Coffea.

Capsicum acts four to eight days.
Antidotes: Calad., Camph., China, Cina., Sulph.
Caps. antidotes: Calad., China, Coff.
Phlegmatic, awkward, easily offended; indolent, melancholic, lack of reaction; dread of open air, lazy, fat, unclean; light hair, blue eyes.

**Carbo-an.** Acts over thirty-six days.
Antidotes: Ars., Camph., Nux-vom., Vin.
Useful in elderly people, with venous plethora, blue cheeks and lips; young scrofulous subjects.

**Carbo-veg.** Acts over thirty-six days.
Antidotes: Ars., Camph., Coff., Lach., Nitr-spr-dulc.
Carbo-v. antidotes: effects of putrid meat or fish, rancid fats; Chin., Lach., Merc.
Low vital powers, venous system predominant; old people; children after exhausting diseases.

Complementary to Kali-c.

**Causticum.** Acts over fifty days.

Antidotes: Asafœt., Coff., Coloc., Nux-v., Nitr-spr-dulc.

Caust. antidotes: Mercur., Sulph.

Inimicals: Acids, Coffea, Phosphor.

Persons with dark hair, rigid fibre. Children with delicate skin.

Cepa; Antidotes: Arnica, Chamom., Veratr.

Cepa is complementary to: Phos., Puls., Sarsap.

Chamomilla. Acts for several days.

Antidotes: Acon., Alum., Borax, Camph., Coccul., Coff., Coloc., Ignat., Nux-v. especially. Puls.

Chamom. antidotes: Coff., Opium.

Complementary to Magnes.

Children, light-brown hair, nervous, excitable temperament. Adults and aged persons with arthritic or rheumatic diathesis.

Chelidonium. Acts over fourteen days.

Chel. follows well after Ledum.

Antidotes: Acon., acids, wine, coffee, Camph.

Chel. antidotes: Bry.

Spare subjects, disposed to abdominal plethora, cutaneous diseases, catarrhs or neuralgia. Blondes.

China-off. Acts two or three weeks.

Antidotes: Aran-diad., Ars., Arn., Bell., Calc-c., Carbo-veg., Eupator-perf., Fer., Ipec., Lach., Mercur., Natr-m., Nux-v., Puls., Sep., Sulph., Veratr.

China antidotes: Ars., Ipec.

Complementary to Ferrum.

Inimical to Selen.

Swarthy persons; debilitated, broken down from exhausting discharges.

Women after menopause; pleurisy, dropsy.

*Cicuta-vir.* Acts five to six weeks.

Antidotes: Arn., Opium; tobacco against massive doses.

Cicuta antidotes: Opium.

Cimicifuga.

Antidotes: Acon., Baptis., Cauloph., Gels., Puls.

Climacteric years; nervousness from anxiety and over-exertion; rheumatic persons, etc.

Cina. Acts two to three weeks.

Antidotes: Camph., China, Capsicum, Ipec., Piper-nigr.

Cina antidotes: Caps., China, Merc.

**Clematis-erecta.** Acts five weeks.

Antidotes: Bryon., Camph.

Torpid, cachectic conditions; light hair.

Cocculus. Acts eight to fourteen days.

Antidotes: Camph., Cham., Cupr., Ignat., Nux-v.

Cocculus antidotes: Alcohol, Cham., Cupr., Ignat., Merc., Nux-v.

Inimical: Coffea.

Coffea. Acts one to two days.

Antidotes: Acon., Cham., Ignat., Nux-v., Puls.

Coff. antidotes: Cham., Coloc., Nux-v., Psorin.

Inimicals: Canth., Caust., Coccul., Ignat.

*Colchicum-autum.* Acts three to four weeks.

Antidotes to poisoning: Amm-caust., a few drops in sugar-water; Bell., Camph., Coccul., Nux-v., Puls.

Gout in persons of vigorous constitutions.

**Colocynthis.** Acts thirty to forty days.

Antidotes: Camph., Coff., Staph., Caust., Chamom. To large doses, tepid milk, Camph., Opium.

Coloc. antidotes: Caustic.

**Conium-mac.** Acts thirty to thirty-five days.

Antidotes: Coff., Nitr-ac., Nitr-spr-dulc.

Conium antidotes: Nitr-acid.

Old men; old maids.

Women with tight, rigid fibre, and easily excited, as also those of opposite temperament. Light-haired persons. Children with marasmus, with frequent sour evacuations worse at night, better during the day.

Crocus-sat. Acts over eight days.

Antidotes: Acon., Bell., Opium.

Croton-tig. It antidotes Rhus poisoning.

*Cuprum-met.* Acts two to three weeks.
Antidotes: Sugar or white of egg for large doses; Hepar-s-c. or potash soap, for poisoning from food containing copper; the aggravation from Cuprum is better from smelling of Camphor $\theta$.
Dynamic antidotes: Bell., Chin., Conium, Dulc., Hepar, Ipec., Merc., Nux-v.
Cupr. antidotes: Aur., Opium.
Complementary to: Calc.-carb.

Cyclamen. Acts two to three weeks.
Antidotes: Camph., Coff., Puls.

**Digitalis.** Acts over six weeks.
Antidotes to large doses: Sweet milk with fœnum græcum (Trigonella fœnum græcum—"Fœnugreek seed"), vegetable acids, vinegar, infusion of galls, Ether, Camph.
Antidotes to small doses: Nux-vom., Opium.
China increases anxiety produced by Digitalis.

Drosera. Acts two to three weeks.
Antidote: Camph.
Sulph. and Veratr. are the most appropriate intercurrents in whooping-cough.
Complementary: Nux-vom.

**Dulcamara.** Acts thirty to forty days.
Antidotes: Camph., Cupr., Ipec., Mercur.
Dulcamar. antidotes: Cupr., Mercur.
Complementary: Baryta-carb.
Incompatible: Bell., Laches.
Phlegmatic, torpid, scrofulous persons, who are restless and irritable, susceptible to changes of weather and taking cold easily.

**Euphorbia-off.** Acts seven weeks.
Antidote: Camph. Succus-citri.

Euphrasia. Acts three to four weeks.
Antidotes: Camph., Puls.

*Ferrum-met.* Acts four to six weeks.
Antidotes: Ars., Chin., Hep., Ipec., Puls., Veratr.
Fer. antidotes (Cupr., Mercur., Prussic-ac.): Ars., Iod., Chin.

Complementary: Alum., Chin.

Persons who, though weak and nervous, have a very red face. Delicate, chlorotic women. Sanguine, choleric people.

Gelsemium.

Antidotes: China, Coff., Natr-mur.

Glonoinum.

Antidotes: Acon., Camph., Coff., Nux-v.

**Graphites.** Acts forty to fifty days.

Antidotes: Acon., Ars., Nux-v., Wine.

Graphites antidotes: Ars., Iod., Rhus-tox.

Complementary: Ars., Caust., Fer., Hepar.

**Guaiacum-off.** Acts over five weeks.

Antidote: Nux-vom.

Guaiac. antidotes: Caust., abuse of Mercur. in rheumatism, gout, contraction.

Syphilides, old women, dark hair and eyes.

Hamamelis.

Antidote: Puls.

Complementary to Ferr. (hemorrhages).

Helleborus. Acts three to four weeks.

Antidotes: Camph., China.

During dentition brain symptoms, weakly, scrofulous children.

**Hepar-s-c.** Acts over eight weeks.

Antidotes: Acetic-acid, Bell., Cham., Silicea.

Hepar antidotes: Potass-iod., Iod., Mercurial and other metallic preparations.

Hydrastis.

Antidote: Sulph.

Hydrastis antidotes: Mercur., Kali-chlor.

Hyos-nig. Acts eight to fourteen days.

Antidotes: Acetic-ac., Bell., Citri-ac., China, Stram.

Hyos. antidotes: Bell., Plumb., Stram., Ether.

Sanguine temperament; nervous, irritable, excitable, hysterical subjects; drunkards, old men, children.

Hypericum.
Antidotes: Arsen., Chamom., Sulph.

Ignatia. Acts five to nine days.
Antidotes: Arnica, Camph., Cham., Coccul., Coff., Nux-v., Puls.
Ignat. antidotes: Zinc., Coff., Cham., Brandy, Puls., Tobacco.
Inimicals: Coff., Tabac.
Suitable to nervous, hysterical females of mild, but easily excited nature.

**Iodium.** Acts over six weeks.
Antidotes: Starch or wheat flour, beat up in water; Ant-tart., Ars., Bell., Camph., Chin., Chin-sulph., Coff., Hepar, Opium, Phos., Spong., Sulph.
Iod. antidotes: Argent-nit., Ars., Calc-c., Merc.
Complementary: Lycopodium.
Suitable particularly to persons with dark eyes and hair; overgrown boys with weak chests; scrofulous diathesis; old people.

Ipecac. Acts twelve to twenty-four hours.
Antidotes: Arn., Ars., Chin., Nux-v., Tabac.
Ipec. antidotes: Alum., Arn., Ars., Chin., vapors of copper, Dulcam., Ferr., Laurocer., Op., Tabac., Tart-emet.
Complementary: Cuprum.

Kali-bichrom.
Antidotes: Ars., Lach., Puls.
Complementary: In dysentery; after Canth. has removed the scrapings, Kali-bi. will often complete the cure.
Fat, light-haired persons, fat, chubby children.

**Kali-carb.** Acts over six weeks.
Antidotes: Camph., Coff., Nitr-spr-dulc.
Complementary to Carb-veg.
Suitable for the aged, rather obese lax fibre; dark hair.
After loss of fluids or vitality; especially in anæmic persons.

Kreosotum.
Antidotes: Acon., Nux-v., Ars., Chin., Ipec.
Inimical: after Carb-veg.

Young people, tall for their age; dark complexion, slight, lean. Complexion livid, disposition sad, irritable; often indicated for old women.

*Lachesis.* Acts four to five weeks.

Antidotes: Ars., Bell., Merc., Nux-v., Phos-ac., heat, alcohol, salt.

Complementary: Lycopodium.

Useful in women during climacteric period.

Laurocerasus. Acts four to eight days.

Antidotes: Camph., Coff., Ipec., Opium.

Painlessness with the ailments.

*Ledum-palustre.* Acts three to four weeks.

Antidote: Camph.

Led. antidotes: Chin., Alcohol, bee stings.

**Lycopodium-clav.** Acts forty to fifty days.

Antidotes: Acon., Camph., Caust., Cham., Coff., Graph., Puls.

Lycopod. antidotes: Chin., but follows well after Calc. or Lachesis.

Complementary to Iodium.

Often useful in old women, persons of keen intellect, but feeble muscular development; lean and predisposed to lung and hepatic affections.

**Magnes-carb.** Acts forty to fifty days.

Antidotes: Chamom., Rheum.

Mag-c. in large doses antidotes Acetic acid.

Complementary to Chamom.

Nervous, irritable temperament; children.

**Magnes-mur.** Acts forty to fifty days.

Antidotes: Camph., Chamom.

Women: especially hysterical, with uterine affections. Children during dentition.

**Manganum-acet.** Acts over forty days.

Antidote: Coffea.

Marum-verum. Acts two to three weeks.

Antidote: Camph.

Menyanthes. Acts two to three weeks.

Antidote: Camph.

**Mercurius.** Acts two to three weeks (Antisyphilitic).
Antidotes: Hepar, Kali-hydr., Nitr-ac., Aurum, Carb-veg., Mezer., Sulph., Iod., Guaiac., Dulcam., Chin., Staphis., Ferr., Bell., Laches., Calc-c., Opium.
Mercur. and Silicea do not follow each other well.

Mercur-cor.
Antidote: Silicea.

**Mezereum.** Acts forty-five to fifty days.
Antidotes: Calc-c., Nux-v., Mercur., Camph.
Mezer. antidotes: Mercur., Nitr-ac., Phosph.
Phlegmatic temperament.

Moschus. Acts one day.
Antidote: Camph.

**Muriatic acid.** Acts over five weeks.
Antidotes to large doses: Carbonate of Soda, Potassa, Lime or Magnesia; small doses: Camph., Bry.
Mur-acid antidotes: Opium and cures muscular weakness from excessive use of Opium.

**Natrum.** Acts thirty to forty days.
Antidote: Camph.

Natrum-carb.
Antidotes: Camph., Nitr-spr-dulc.
Natr-carb. antidotes: China.

**Natrum-mur.** Acts forty to fifty days.
Antidotes: Nitr-spr-dulc., Phos., Arsen.,
Natr-mur. antidotes: Argent-nitr., Chin-sulph., bee stings.
Complementary: Apis.

**Nitrum.** Acts over six weeks.
Antidote: Nitr-spr-dulc.
Camph. increases the pains.

**Nitric acid.** Acts for over forty days.
Antidotes: Calc-c., Camph., Hep., Merc., Mez., Sulph.; Alkalies, Soap, Magnesia.
Nitr-acid antidotes: Calc-c., Digit., Merc.
Complementary: Calad.
Inimical: Lachesis.
Nitr-ac. is especially active after Kali.

**Nux-mosch.** Acts six to eight days.

Antidotes: Semen-capi, Gels., Lauroc., Nux-v.

Nux-mosch. antidotes: Ars., Rhodo., Lauroc., inhalations of Mercury, lead colic.

Suits mostly women and children and the aged.

**Nux-vom.** Acts ten to twelve days.

Antidotes: Acon., Camph., Cham., Coccul., Coff., Puls., Wine, Alcohol.

Nux-vom. is an antidote to: abuse of aromatics, drastics, hot medicines, narcotics, bad effects of coffee and alcoholic drinks.

Complementary: Sulph.

Inimical: Zinc.

Suitable for thin, irritable, choleric persons with dark hair, who make great mental exertions or lead a sedentary life. Debauchers, who are irritable and thin.

*Oleander.* Acts three to four weeks.

Antidote: Camph.

Opium. Acts only a few hours.

Antidotes: Strong Coffee, Bell., Ipec., Nux-v., Vinum, Vanil-arom.

Opium antidotes: Bell., Digit., Lach., Merc., Nux-v., Strychnia, Plumb., Stram., Tart-emet.

Especially suitable for children and old persons. Frequently suited to persons addicted to liquors.

Paris quadrifol. Acts two to four days.

Antidote: Camph., Coff.

**Petroleum.** Acts forty to fifty days.

Antidote: Nux-vom.

Petrol. antidotes: Lead poisoning.

**Phosphorus.** Acts over forty days.

Antidotes: Nux-v., Coff., Tereb., Camph., Vinum.

Phos. antidotes: Tereb., Rhus-ven.

Complementary: Cepa, Ars.

Inimical: Causticum.

Tall, slender (slim) women, disposed to stoop. Nervous, weak; grow too rapidly.

**Phosphoric acid.** Acts over forty days.
Antidotes: Camph., Coff.
Bad effects from growing too rapidly, as if beaten in back and limbs.

Phytolacca decandra.
Antidotes: milk and salt; Ignat., Sulph., Opium.

**Platina.** Acts five to six weeks.
Antidotes: Puls., Nitr-spr-dulc.
Platina antidotes bad effects of lead.
Especially suited to women with dark hair, rigid fibre.

*Plumb.* Acts three to four weeks.
Antidotes: Alumen, Alum., Opium, Petrol., Nux-vom., Platina, Anti-crud., Coccul., Zinc.
[Alcohol may be used as a preventive.]

Podophyl-pelt.
Antidotes: Lactic-ac., Nux-vom.
Complementary: Salt.
Bilious temperament, especially after mercurialization.

Psorinum.
Antidote: Coff.
Scrofulous; nervous, restless, easily startled.
Psoric constitutions; especially when other remedies fail to permanently improve.
Lack of reaction after severe diseases.
Pale, sickly, delicate children.

*Pulsatilla.* Acts eight to fourteen days.
Antidotes: Chamom., Coff., Ignat., Nux-vom.
Puls. antidotes: Chin., Ferr., Sulph., Sulph-ac. Vapor of Mercury or Copper, Coff., Cham., Bell., Colch., Lycop., Platin., Stram., Sabad., Ant-tart.
Complementary to Puls.: Lycop., Sulph-ac.
Sandy hair, blue eyes, pale face, inclined to grief and submissiveness; easily moved to tears or laughter. Often indicated with women and children.

*Ranunculus-bulb.* Acts four to six weeks.
Antidotes: Bry., Camph., Puls., Rhus.
Inimicals: Alcohol, Nitr-spr-dulc., Staph., Sulph., Vinegar, Wine.

*Ranunculus-scel.* Acts five to six weeks.
Antidote: Camph.
Rheum. Acts two to three days.
Antidotes: Camph., Cham., Coloc., Merc., Nux-v., Puls.
Complementary: Magnes-carb.
**Rhododendron.** Acts five to six weeks.
Antidotes: Bry., Clemat., Rhus, Camph.
*Rhus-tox.* Acts three to six weeks.
Antidotes: Bell., Bry., Camph., Coff., Crot-tig.. Sulph.
Rhus antidotes: Bry., Ranunc., Rhododendron, Tart-emet.
Complementary: Bry.
Inimical: Apis.
Ruta-graveolens. Acts eight to fourteen days.
Antidote: Camph.?
Ruta antidotes: Mercur.
*Sabadilla.* Acts three to four weeks.
Antidotes: Camph. (?), Puls.
Children; old people. Light hair; muscles lax.
*Sabina.* Acts three to four weeks.
Antidote; Puls.
Chronic ailments of women; arthritic pains, tendency to miscarriage.
Sambucus. Acts three to four hours.
Antidotes: Ars., Camph.
Samb. antidotes: Ars.
Scrofulous children; people formerly fat and robust become emaciated.
After violent emotions, grief, anxiety, or excess in sexual indulgence.
Sanguinaria-can.
It antidotes Rhus-radicans.
**Sarsaparilla.** Acts over five weeks.
Antidotes: Bell., Merc.
Vinegar appears at first to increase the effects of Sarsap.
Secale-cor. Acts two to three weeks.
Antidotes; Camph. (Solan-nig.).
Similar to Ars. but heat and cold act oppositely.
Irritable, plethoric subjects. Women of very lax muscular fibre; feeble, cachectic, thin, scrawny. Old, decrepit persons. Nervous temperament.

*Selenium.* Acts five to six weeks.

Antidotes: Ignat., Puls.

Incompatible: Chin., Wine.

**Senega.** Acts over four weeks.

Antidotes: Arnic., Bell., Bry., Camph.

**Sepia.** Acts forty to fifty days.

Antidotes: Vegetable acids, inhalations of Nitr-spir-dulc. is the most powerful antidote; Anti-crud., Anti-tart., Acon.

Sepia antidotes: Calc-c., Chin., Merc., Phos., Sarsap., Sulph.

Incompatible: Lachesis.

Especially suited to persons with dark hair, for women and particularly during pregnancy, in child-bed and while nursing.

**Silicea.** Acts forty to fifty days.

Antidotes: Fluor-acid., Hep., Calc-s-c., Camph.

Silicea antidotes: Sulph., Mercur., but does not follow the potentized Merc. well.

Complementary: Thuja.

Especially suitable for children with large heads, open sutures; much sweat about the head; large abdomen.

Nervous irritable persons, with dry skin, profuse saliva, diarrhœa, night-sweats.

Weakly persons, fine skin, pale face, light complexion; lax muscles.

Scrofulous diathesis.

Rachitic, anæmic conditions: caries, over-sensitive, imperfectly nourished from imperfect assimilation.

Stone-cutters; chest affections, and total loss of strength.

*Spigelia.* Acts three to four weeks.

Antidotes: Aur., Coccul., Puls., Camph.

**Spongia.** Acts three to four weeks.

Antidote: Camph.

Squilla. Acts two to three weeks.

Useful after Bry.

**Stannum.** Acts over five weeks.

Antidote: Puls.

Stan. follows well after Causticum.

Complementary: Puls.

*Staphisagria.* Acts three to four weeks.

Antidote: Camph.

Staph. antidotes: Merc., Thuja.

Incompatible: Ran-bulb.

Stramonium. Acts one-half to one day.

Antidotes: Bell., Hyos., Nux-v.; against large doses: lemon-juice, senna, tobacco injections, vinegar.

Stramon. antidotes: ailments from vapor of Mercury, Plumb.

Suitable for children, especially in chorea, mania, fever. Young, plethoric persons.

**Strontiana.** Acts over forty days.

Antidote: Camph.

**Sulphur.** Acts forty to fifty days.

Antidotes: Acon., Camph., Cham., Chin., Merc. (Nux-v.), Puls., Rhus-tox., Sepia.

Sulph. antidotes: Chin., Iod., Merc., Nitr-ac., Rhus-tox., Sepia; ailments from the use of metals generally.

Complementary: Aloe-soc.

Sulph. is especially suited for lean, stoop-shouldered persons. It frequently serves to rouse the reactive power of the system, when carefully selected remedies have failed to produce a favorable effect, especially in acute disease.

**Sulph-acid.** Acts over four weeks.

Puls. is an antidote and also complementary.

Sulph-ac. antidotes the bad effects of lead-water.

Frequently indicated for old people, particularly women. Light-haired people.

Flushes of heat in climacteric years.

Tabacum.

Antidotes: Ars., Ipec., Nux-v., Phos., Ignat., Puls., Clemat., Sepia, Lycopod.

Plantago-maj. has often caused an aversion to tobacco.

Taraxacum. Acts two to three weeks.

Antidote: Camph.?

Terebinth. is antidoted by Phos.

**Thuja.** Acts three weeks.

Antidotes: Cham., Coccul., Camph., Merc., Puls., Sulph.
Thuja antidotes: Iod., Merc., Nux-v., Sulph., Thea.

Valeriana. Acts eight to ten days.
Antidotes: Camph., Coff., Puls.
Valerian. antidotes: Cham.
Nervous, irritable, hysteric individuals.

Veratrum-alb. Acts five to eight days.
Antidotes: Acon., Camph., Chin., Coff.
Veratr. antidotes: Ars., Chin., Cuprum, Fer., Op., Tobacco.
Lean, choleric, or melancholic persons; Anæmia; Children.

Veratrum-vir.
It antidotes spasms from Strychnine.
Full-blooded, plethoric persons.

Verbascum. Acts four to eight days.

Viola-odorata. Acts two to four days.
Antidote: Camph.

Viola-tri. Acts eight to fourteen days.
Antidote: Camph.

Zincum. Acts thirty to forty days.
Antidotes: Hepar, Ignat., Camph.
Incompatible: Cham., Nux-v.

